# Persuasive System Design Does Matter: A Systematic Review of Adherence to Web-Based Interventions

**DOI:** 10.2196/jmir.2104

**Published:** 2012-11-14

**Authors:** Saskia M Kelders, Robin N Kok, Hans C Ossebaard, Julia EWC Van Gemert-Pijnen

**Affiliations:** ^1^Center for eHealth Research and Disease ManagementDepartment of Psychology, Health and TechnologyUniversity of TwenteEnschedeNetherlands; ^2^National Institute for Public Health and the EnvironmentBilthovenNetherlands; ^3^EMGO Institute for Health and Care ResearchDepartment of Clinical Psychology, Faculty of Psychology and EducationVU UniversityAmsterdamNetherlands

**Keywords:** Systematic review, web-based interventions, adherence, attrition, persuasive technology, behavior change

## Abstract

**Background:**

Although web-based interventions for promoting health and health-related behavior can be effective, poor adherence is a common issue that needs to be addressed. Technology as a means to communicate the content in web-based interventions has been neglected in research. Indeed, technology is often seen as a black-box, a mere tool that has no effect or value and serves only as a vehicle to deliver intervention content. In this paper we examine technology from a holistic perspective. We see it as a vital and inseparable aspect of web-based interventions to help explain and understand adherence.

**Objective:**

This study aims to review the literature on web-based health interventions to investigate whether intervention characteristics and persuasive design affect adherence to a web-based intervention.

**Methods:**

We conducted a systematic review of studies into web-based health interventions. Per intervention, intervention characteristics, persuasive technology elements and adherence were coded. We performed a multiple regression analysis to investigate whether these variables could predict adherence.

**Results:**

We included 101 articles on 83 interventions. The typical web-based intervention is meant to be used once a week, is modular in set-up, is updated once a week, lasts for 10 weeks, includes interaction with the system and a counselor and peers on the web, includes some persuasive technology elements, and about 50% of the participants adhere to the intervention. Regarding persuasive technology, we see that primary task support elements are most commonly employed (mean 2.9 out of a possible 7.0). Dialogue support and social support are less commonly employed (mean 1.5 and 1.2 out of a possible 7.0, respectively). When comparing the interventions of the different health care areas, we find significant differences in intended usage (p = .004), setup (p < .001), updates (p < .001), frequency of interaction with a counselor (p < .001), the system (p = .003) and peers (p = .017), duration (F = 6.068, p = .004), adherence (F = 4.833, p = .010) and the number of primary task support elements (F = 5.631, p = .005). Our final regression model explained 55% of the variance in adherence. In this model, a RCT study as opposed to an observational study, increased interaction with a counselor, more frequent intended usage, more frequent updates and more extensive employment of dialogue support significantly predicted better adherence.

**Conclusions:**

Using intervention characteristics and persuasive technology elements, a substantial amount of variance in adherence can be explained. Although there are differences between health care areas on intervention characteristics, health care area per se does not predict adherence. Rather, the differences in technology and interaction predict adherence. The results of this study can be used to make an informed decision about how to design a web-based intervention to which patients are more likely to adhere.

## Introduction

Web-based interventions for promoting health and health-related behaviors are seen in many variations and health care areas. According to Barak et al. [1] a web-based intervention is:

...a primarily self-guided intervention program that is executed by means of a prescriptive online program operated through a website and used by consumers seeking health- and mental health–related assistance. The intervention program itself attempts to create positive change and or improve/enhance knowledge, awareness, and understanding via the provision of sound health-related material and use of interactive web-based components.

A web-based intervention can involve therapy that lasts for a predetermined, fixed period of time. However, it can also be a continuous program with no specific end date that supports self-management among patients with a chronic condition. It is made up of different, inseparable aspects which, according to Barak et al [1], are as follows: program content, multimedia choices, interactive online activities, and guidance and supportive feedback.

Evidence exists to support the effectiveness of web-based interventions. Research has shown these interventions to be effective in different areas of health care [2-7]. However, many evaluations of eHealth interventions report either no positive effects at all or only limited ones [8-12]. One of the issues that is frequently addressed is the problem of non-adherence [11, 13-17], which refers to the fact that not all participants use or keep using the intervention in the desired way. Research suggests that non-optimal exposure to the intervention lessens the effect of these interventions [18, 19]. Gaining an insight into the factors that influence adherence should therefore be one of the main focus areas in any research study into web-based interventions. In this context, it is important to stress the difference between the terms “adherence” or “non-usage attrition” and “dropout.” Dropout, or dropout attrition, refers to participants in a study who do not fulfill the research protocol (eg, filling out questionnaires). This is not a focus area of this study. Adherence, or non-usage attrition, refers to the extent to which individuals experience the content of an intervention [13, 15]. This is the focus of our study.

When looking at literature about adherence to a therapeutic regimen [20, 21], adherence is seen as the extent to which the patient’s behavior matches the recommendations that have been agreed upon with the prescriber. The term is often seen as a reaction to the term “compliance,” which has a more coercive connotation. Consequently, in adherence, the patient plays an active role in achieving this behavior [21]. At the same time, there is also a norm or recommendation from a prescriber, which the patient tries to match. This recommendation is missing from the definitions of both adherence and non-usage attrition [13, 15]. In this study, we elaborate on the definition by introducing the concept of “intended usage.” Intended usage is the extent to which individuals *should* experience the content (of the intervention) to derive maximum benefit from the intervention, as defined or implied by its creators. This matches the norm or recommendation from the definition of adherence to a therapeutic regimen. By comparing the observed usage of an individual to the intended usage of a web-based intervention, we can establish whether or not this individual adheres to the intervention. In this context, adherence is a process that cannot be assessed solely by measuring usage at the beginning and end of the intervention. Rather, it has to be assessed throughout the entire process to establish whether or not an individual adheres to the intervention at each and every step of the way. Finally, by comparing the observed usage of each individual to the intended usage of the web-based intervention, the percentage of individuals that adhere to the intervention can be calculated. This results in a more objective measurement of adherence, which can then be compared to other interventions, even if the intended usage is different.

Adherence to web-based interventions has been the subject of research for some time. Many studies focus on whether and which respondents’ characteristics can explain variations in adherence [11, 13, 16, 22]. Although this is a very important line of study, it seems to take the technology of web-based interventions for granted. Technology as a means to communicate the content has been neglected in research. Indeed, this technology is often seen as a black box: a mere tool that has no effect or value and serves only as a vehicle for the delivery of intervention content. In line with a recent viewpoint paper, we propose to examine the technology from a holistic perspective and see it as a vital and inseparable aspect of the web-based intervention [12]. This approach has been recommended in recent literature [10, 11, 13, 23] and has been the key point in the field of persuasive technology [24], where there are examples of studies on the persuasive capacities of technology to support web-based interventions in the health care domain [25-28].

Recently, two systematic reviews on the influence of intervention factors on adherence to web-based interventions were published [29, 30]. Although both reviews provide valuable insights, we feel that there are shortcomings that limit the applicability of these results for our objectives. First, with regard to adherence, the study of Brouwer [29] takes exposure to interventions delivered via the internet as the outcome measure. Exposure is seen as the number of times the user or patient logged on, the time spent on site, page views, etc, but these are static measurements unrelated to the usage intended by these interventions. This gives limited insights into the process of usage and adherence, which makes it difficult to compare different interventions and specify how well certain interventions are doing. A review by Schubart [30] fails to distinguish between dropout and adherence. This approach limits the applicability of the results because, in real-life implementation of web-based interventions, there is no research protocol to adhere to, only the intervention. The results of Schubart’s review [30] cannot be generalized to these situations because we do not know whether engagement is due to the research or the intervention.

Furthermore, regarding the intervention factors, both studies use an ad hoc classification of these factors without a theoretical foundation, which makes it difficult to generalize and explain the results. We consider a web-based intervention as consisting of content, interaction, and technology. And, although these aspects are inseparable, they can be looked at in a structured manner. Both earlier reviews use a classification that, in our opinion, has substantial overlap in the goals to be achieved with these aspects. For example, in the review by Brouwer [29], a distinction is made between interactive behavior change strategies and interactive elements. It is stated that the goal of interactive elements is to “improve the attractiveness of the intervention or to provide the option for more information,” but this is not mutually exclusive with interactive behavior change strategies. For example, a quiz is seen as an interactive element, but in our opinion it can also be used as a means of receiving tailored feedback or as a way to self-monitor your knowledge or behavior. Allocating a quiz to one of these categories is therefore problematic. The categorization of intervention factors in the review by Schubart [30] lacks depth and tries to encompass in one single categorization both modality (ie, the channel through which content is delivered; for example, email or telephone) and strategy (eg, feedback).

The current study attempts to overcome these shortcomings by employing a more objective and comparable measurement of adherence to web-based interventions and a classification of technology based on persuasive technology literature.

From the field of persuasive technology we learn that technology has the capacity to be persuasive through its role as a tool, a medium, and a creator of experiences [24]. Fogg’s definition of persuasive technology limits this field to human-computer interaction and does not include computer-mediated communication (ie, including interaction with a person). However, we feel that it is unnecessary and undesirable to separate these two aspects of technology, particularly in the area of health care, because a web-based intervention is made up of different, inseparable aspects. We therefore propose a broader application of the term “persuasive technology” to include both human-computer interaction and computer-mediated communication. Accordingly, regarding the aspects of a web-based intervention, we propose a more pragmatic conceptual division between technology (ie, all the features of the web-based intervention, including multimedia and online activities) and interaction (ie, all interactions between the user or patient and the intervention, a counselor, or peers), which is slightly different from the aspects proposed by Barak. Following Fogg’s work, Oinas-Kukkonen introduces a framework to classify technology in its persuasive functions [31]. This persuasive system design (PSD) model, which is used, for example, in a study by Lehto and colleagues [32], classifies features of the technology as primary task support, dialogue support, social support, and credibility support. By applying this model to web-based interventions, we can systematically look at how persuasive system design categories are used and investigate their possible influence on adherence.

This study investigates whether intervention characteristics and persuasive design affect adherence to a web-based intervention. Web-based interventions are applied in various health care domains and intuitively it seems that there are differences between web-based interventions aimed at people with a chronic condition, at lifestyle change, or at mental health, because of the target group, involvement with a health care professional, and duration of the interventions. However, the underlying principles may well be the same. Therefore, from an intervention perspective, there is no absolute need to see these areas as being so different from each other that they cannot be compared. Consequently, it is interesting to see whether the preconceptions about the differences can be confirmed and whether there is added value for researchers and designers in one area to look at interventions from a different area.

Our systematic review aims to answer the following research questions: (1) What are the key characteristics of web-based interventions in terms of technology and interaction? (2) Are there any differences in intervention characteristics between web-based interventions aimed at chronic conditions, lifestyle, or mental health? (3) What percentage of participants adhere to web-based interventions? (4) Which characteristics of web-based interventions related to technology and interaction are linked to better adherence? These insights can help us understand and reduce the impact of non-adherence.

## Methods

### Search Strategy

**Figure 1:**
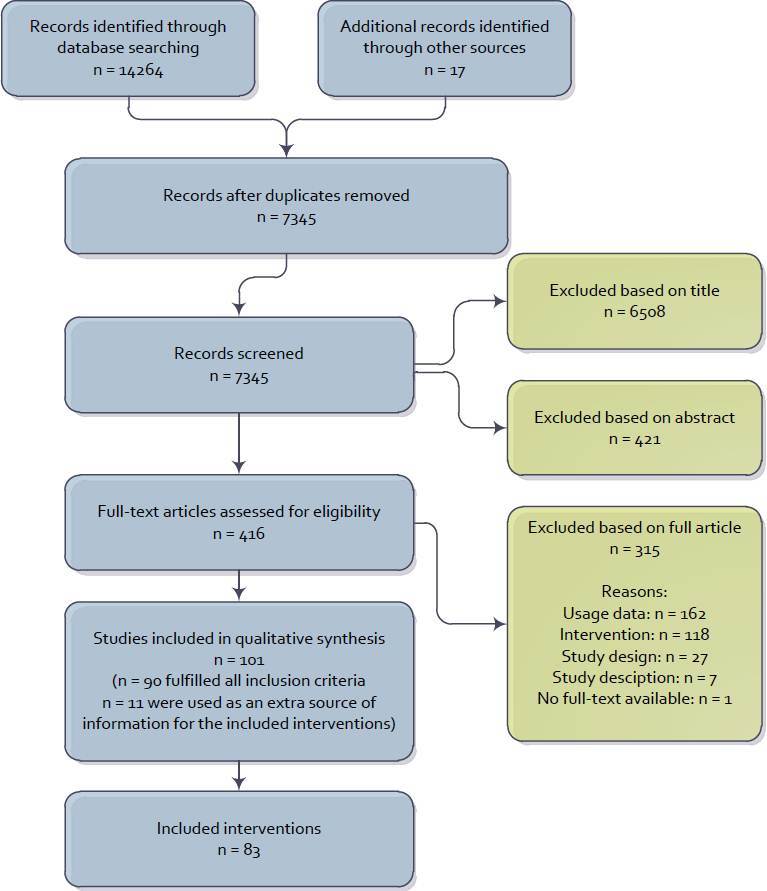
Flow diagram of study selection.

We conducted a comprehensive literature search using the following bibliographic databases: Web of Knowledge, EBSCOhost, PiCarta, SciVerse Scopus, and ScienceDirect. We used a combination of the constructs “web-based,” “intervention,” “adherence,” and “health.” For each construct, we used several keywords (see Multimedia Appendix 1) to ensure a broad coverage of published studies in our review. Following this search strategy, we identified 14,264 articles published up to 2011 Oct 26 (see Figure 1 for the full flow diagram of article selection).

### Eligibility Criteria

The review is limited to studies of web-based interventions in the health care domain. The criteria used for including a study were: (1) it involved a web-based intervention for promoting health through behavioral change; (2) the web-based intervention was intended to be visited and used on more than one occasion; (3) the research included an assessment of the effect of the intervention; (4) the study reported objective, quantifiable measurements of usage for the intervention; and (5) the study was published in either English or Dutch. Exclusion criteria were as follows: (1) dropout attrition and non-adherence were indistinguishable; (2) the intervention was aimed at care providers or relatives of the “patient;” (3) the description of the intervention did not include information about the applied persuasive features of the technology; and (4) the web-based intervention was not primarily intended to be used through a computer or laptop at the user’s or patient’s home. In addition, we only included peer-reviewed, published articles.

### Study Selection and Data Collection

The study selection was done in three steps. First, the titles of all retrieved articles were screened for eligibility by two authors (SK and RK). Second, the abstracts of all initially relevant articles were screened for eligibility by the same authors. Finally, the full text of all remaining publications was checked for inclusion by two authors (SK and RK or SK and JvG). In cases where the suitability of a study came into question during one of the steps, it was included in the next step. Disagreements about including the full text publication were discussed until agreement was reached. To check whether any eligible publications had been overlooked during the initial search process, the reference lists of all systematic reviews that were identified in the original search were checked to find additional publications that met our inclusion criteria.

The characteristics of all of the interventions that were included were coded by two researchers (SK and RK) using a data extraction form based on a protocol for the systematic review of eHealth technologies [33]. Where possible, data was extracted using the CONSORT-EHEALTH checklist [12]. For the extraction, we relied on information that was available in the published literature. The basis of the data extraction was the intervention, not the study itself. This meant that for some interventions data from more than one article was used. Furthermore, when a study described more than one web-based intervention (eg, a comparison of two web-based interventions), all web-based interventions were coded separately.

### Data Items

The following characteristics were coded:

#### Intervention Name

The name of the intervention was recorded. If the intervention had no name, the intervention was named after the first author of the primary article about the intervention.

#### Behavior or Condition

The targeted behavior or condition of each intervention was recorded. Furthermore, we recorded the area of health care targeted by the intervention (chronic condition, lifestyle, or mental health).

#### Studies and Study Design

For each intervention, the studies that were used to code the characteristics of the intervention were recorded. Furthermore, we also recorded whether these studies were randomized controlled trials (RCTs) or observational studies without randomized control groups.

#### Intended Usage

Intended usage was defined as the extent to which the developers of the intervention felt that the intervention should be used to achieve the desired effect ([12] 5ix). When this information was not reported, it was inferred from the description of the intervention. For example, interventions requiring patients to monitor their behavior and receive feedback once a week to achieve the desired effect were coded as intended to be used once a week.

#### Actual Usage

All reported information regarding the usage of the intervention (related to its intended usage) was collected, including the number of times the user or patient logged on and the number of modules completed ([12] 6aii).

#### Adherence

A percentage of adherence was calculated to enable us to compare the different interventions. We did this by calculating the percentage of participants that adhered to the intervention. For example, when the intended use of an intervention was “complete 8 modules” and 60 out of 100 participants completed 8 modules, the adherence was 60%. For each intervention that was included, we calculated one overall adherence percentage. When more studies about the same intervention yielded different adherence percentages, we calculated the overall adherence percentage using a weighted average, based on the number of participants in each study. Furthermore, when the study included a waiting list and the respondents in this waiting list received access to the intervention at a later stage, the adherence was calculated based on usage data for all participants, including the waiting list group.

#### Updates

The frequency of content updates for the web-based intervention for a participant was recorded. This could be based on new information being uploaded for all participants or on a new lesson becoming available for a specific participant.

#### Duration

The duration of the intervention in weeks was recorded.

#### Setup

For each intervention, we created a record indicating whether the setup was modular (ie, content is delivered in a sequential order, whereby new content is made available when the user reaches a certain point) or free (ie, all the content of the intervention is available to the user from the start).

#### Interaction

All information about the interaction with participants was recorded ([12] 5viii, 5x, and 5xi). This interaction could be with the system (eg, automatic email reminders or a web-based automated response to filling out an exercise), with a counselor (eg, through email, telephone, or face-to-face meetings), or with peers (eg, through a discussion board, chat group, or face-to-face group sessions).

#### Modality

We recorded when interaction with the system, counselor, or peers took place through a different modality than web-based (face-to-face meeting, telephone, or SMS). An exception was made when the study protocol included a face-to-face meeting or telephone intake. This was not coded as interaction through a different modality because it was not part of the actual intervention.

#### Persuasive Technology in the Intervention

The applied principles of persuasive technology within the interventions were coded according to the PSD framework of Oinas-Kukkonen and Harjumaa [31]. We omitted system credibility support because of an observed lack of reporting of these principles in the studies that were included. The elements from the PSD framework on primary task, dialogue, and social support, with the definitions and the coding scheme we used, are presented in Table 1. The coding scheme is somewhat modified for the purpose of this study and to account for the computer-mediated communication included. However, when coding the persuasive technology elements, the technology was central, not the content of the interaction. Therefore, when computer-mediated communication was present, the content of this communication was not coded as persuasive technology. For example, when a feedback message from a care provider contained praise, this was not coded as dialogue support. When the technology provided a praising message after the user had successfully filled out a diary entry, then it was coded. For each intervention, the elements that were present were coded, irrespective of whether the designers of the intervention deliberately included these elements as persuasive technology elements. To check for differences in interpretation when coding the persuasive technology elements, 10 interventions were coded by 2 researchers (SK and LvG). The interrater reliability, measured by Cohen’s kappa, was 0.91.

### Analyses

All data on each intervention was entered in SPSS version 19.0 (IBM Corporation, Somers, NY, USA), and we treated each intervention as a separate case. Descriptive data of the combined data of all included interventions on all variables were calculated using SPSS. Differences in variables between health care areas were calculated using Fisher’s exact tests (because of the small expectation values) and one-way analyses of variance. To investigate whether the characteristics of the included interventions could predict the observed adherence, we performed a hierarchical multiple linear regression analysis, using a block-wise “enter” method. The first block was related to the context of the web-based intervention and included the health care area (coded as dummy variables) and the study design (RCT vs observational), which other researchers have proposed to influence adherence or the effect of web-based interventions [7, 29, 34]. The second block relates to our concept of interaction as one of the aspects of a web-based intervention and consists of the frequency of interaction with a counselor, the system, and peers, as well as the modality employed. The third and fourth blocks relate to our concept of technology in a web-based intervention, where the third block contains the intervention characteristics intended usage, setup, updates, and duration, and the last block contains the categories of persuasive system design. It is important to note that we chose to include the categories, and not the separate elements in the multiple regression, because (1) the results could be biased when some elements are hardly used and these elements are entered as predictors; (2) entering all 21 elements increases the chance of a type I error; and (3) the PSD model has grouped the elements on their key benefits (when the benefits of the specific elements in a category are similar, then looking at the specific elements could cause the overall influence of the category to be missed).

**Table 1:** PSD framework elements coding scheme.

Principle and definition according to PSD framework [31]			Coded as element included when the web-based intervention:	Example
**Primary Task Support**				
	Reduction	A system that reduces complex behavior into simple tasks helps users perform the target behavior, and it may increase the benefit/cost ratio of a behavior.	Specifically divides the target behavior into small, simple steps	A web-based intervention for weight management includes a diary for recording daily calorie intake, thereby dividing the target behavior (reducing calorie intake) into small, simple steps of which one is recording calorie intake
	Tunneling	Using the system to guide users through a process or experience provides opportunities to persuade along the way.	Delivers content in a step-by-step format with a predefined order	A web-based intervention for the prevention of depression that delivers the content in sequential lessons that can only be accessed when the previous lesson is completed
	Tailoring	Information provided by the system will be more persuasive if it is tailored to the potential needs, interests, personality, usage context, or other factors relevant to a user group.	Provides content that is adapted to factors relevant to a user group, or when a counselor provides feedback based on information filled out by a participant	A web-based intervention for supporting self-management among patients with diabetes provides information adapted to patients based on whether they have diabetes mellitus type I or II
	Personalization	A system that offers personalized content or services has a greater capability for persuasion.	Provides content that is adapted to one user (ie, the name of the user is mentioned and/or the user can adapt a part of the intervention)	A web-based intervention for increasing physical activity allows users to choose whether they want to see their weekly activity score on the home page or not
	Self-monitoring	A system that keeps track of one’s own performance or status supports the user in achieving goals.	Provides the ability to track and view the user’s behavior, performance or status	A web-based intervention for the treatment of alcohol dependence provides a diary to track and view daily alcohol use
	Simulation	Systems that provide simulations can persuade by enabling users to observe immediately the link between cause and effect.	Provides the ability to observe the cause-and-effect relationship of relevant behavior	A web-based intervention for smoking cessation includes a calculator that shows how much users will save when they quit smoking
	Rehearsal	A system providing means with which to rehearse a behavior can enable people to change their attitudes or behavior in the real world.	Provides the ability and stimulation to rehearse a behavior or to rehearse the content of the intervention	A web-based intervention for supporting self-management in patients with epilepsy starts each lesson with the same important exercise for stress-management
**Dialogue Support**				
	Praise	By offering praise, a system can make users more open to persuasion.	Offers praise to the participant on any occasion	A web-based intervention that aims to promote healthy nutritional habits compliments participants when they have eaten 2 pieces of fruit for 5 days
	Rewards	Systems that reward target behaviors may have great persuasive powers.	Offers some kind of reward when the participant performs a target behavior relating to the use or goal of the intervention	A web-based intervention for the treatment of social phobia gives points to participants when they engage in exposure exercises
	Reminders	If a system reminds users of their target behavior, the users will more likely achieve their goals.	Provides reminders about the use of the intervention or the performance of target behavior	A web-based intervention to support self-management among patients with rheumatic arthritis sends an automatic email message to remind the participant that the new lesson may begin
	Suggestion	Systems offering fitting suggestions will have greater persuasive powers.	Provides a suggestion to help the participants reach the target behavior	A web-based intervention for weight management provides low-calorie recipes
	Similarity	People are more readily persuaded through systems that remind them of themselves in some meaningful way.	Is designed to look familiar and designed especially for the participant	A web-based intervention for the treatment of panic disorder in teenage girls explains the exercises through a teenage girl with panic problems
	Liking	A system that is visually attractive for its users is likely to be more persuasive.	Is visually designed to be attractive to the participants	During the design of a web-based intervention to increase physical activity in middle-aged women, a representative group is asked for feedback on the design and their feedback is subsequently incorporated in the new design
	Social role	If a system adopts a social role, users will more likely use it for persuasive purposes.	Acts as if it has a social role (eg, a coach, instructor, or buddy)	A web-based intervention to support self-management among patients with migraine incorporated an avatar to guide the participant through the intervention
**Social Support**				
	Social learning	A person will be more motivated to perform a target behavior if (s)he can use a system to observe others performing the behavior.	Provides the opportunity and stimulates participants to see others using the intervention or performing the target behavior	A web-based intervention for weight management provides the option, and stresses the importance, of posting physical activity self-monitoring data on the discussion board and commenting on the performance of others
	Social comparison	System users will have a greater motivation to perform the target behavior if they can compare their performance with the performance of others.	Provides the opportunity for participants to compare their behavior to the target behavior of other participants and stimulates them to do this	A web-based intervention for drug abuse prevention for teenagers automatically compares the response of the participant to other users of the intervention
	Normative influence	A system can leverage normative influence or peer pressure to increase the likelihood that a person will adopt a target behavior.	Provides normative information on the target behavior or the usage of the intervention	A web-based intervention to promote self-management among patients with COPD provides feedback on the level of physical activity of the participant by comparing it to the physical activity of well-managed COPD patients
	Social facilitation	System users are more likely to perform target behavior if they discern via the system that others are performing the behavior along with them.	Provides the opportunity to see whether there are other participants using the intervention	A web-based intervention for smoking cessation includes a discussion board for users of the intervention
	Cooperation	A system can motivate users to adopt a target attitude or behavior by leveraging human beings’ natural drive to cooperate.	Stimulates participants to cooperate to achieve a target behavior	A web-based intervention for the promotion of physical activity stimulates participants to form groups and to achieve the group goal of a certain number of steps each week
	Competition	A system can motivate users to adopt a target attitude or behavior by leveraging human beings’ natural drive to compete.	Stimulates participants to compete with each other to achieve a target behavior	A web-based intervention for diabetes management among children includes a leaderboard in which the children who enter blood glucose levels at the right times receive the highest place
	Recognition	By offering public recognition for an individual or group, a system can increase the likelihood that a person/group will adopt a target behavior.	Prominently shows (former) participants who adopted the target behavior	A web-based intervention treatment of anxiety includes a testimonial page where successful users of the intervention tell their story

## Results

### Study Selection

The search yielded 7345 unique titles. After title, abstract, and full-text screening, 101 articles on 83 interventions were included (Figure 1). In total, 315 articles were excluded based on the full text. The most common reason for exclusion was related to usage data: the lack thereof (n = 84) or the presentation of inadequate (ie, subjective or not usable for calculating adherence) usage data (n = 78). Other studies were excluded based on the studied intervention: not aimed at health promotion by changing behavior (n = 40); not primarily meant to be used from a computer or laptop at the user’s home (n = 41); not intended to be visited and used on more than one occasion (n = 34); or not targeted at the patient (n = 3). Twenty-seven publications were excluded because the study design did not include an assessment of the effect of the intervention (eg, when they only presented qualitative data on the design of an intervention) or when the study design did not provide unique usage data (eg, a study about the long-term effects of an intervention). Seven publications were excluded because of the description of the intervention or study: in 4 publications no information could be gathered on the applied persuasive features of the technology from the description of the intervention and in 3 publications the data on the number of participants and their usage of the intervention was unclear. Finally, in the case of one citation, the full text could not be retrieved; this citation was therefore excluded.

### Characteristics of the Studies that Were Included

The 83 interventions that were included are presented in Multimedia Appendix 2. Overall, 19 interventions targeted a specific chronic condition (diabetes was targeted most often with 6 interventions). Sixteen interventions targeted a lifestyle behavior (weight management was targeted most often with 7 interventions). Smoking cessation was also often seen (5 interventions were targeted solely on smoking cessation and 1 intervention included smoking cessation as one of multiple targeted behaviors). Finally, mental health was targeted most often in the studies that were included. Of these 48 interventions, 12 focused on social phobia, although it should be noted that these interventions are only from two research groups that extensively studied their interventions. Depression, panic disorder, and anxiety were also targeted frequently in the interventions that we included (10, 8 and 7 interventions, respectively).

Table 2 presents an overview of the variables of the interventions that were coded and their distribution over the different areas (chronic condition, lifestyle, and mental health). Overall, we can see that most interventions were meant to be used once a week, were set up in a modular way, were updated once a week, and lasted for approximately 16 weeks (median duration 10 weeks). Face-to-face, telephone, and SMS support, or a combination of these modes, were infrequently used, with 4 interventions combining face-to-face and telephone support (interventions 3, 10, 33, and 72) and 2 interventions combining telephone and SMS support (interventions 24 and 81). Seventy-six per cent of the interventions included interaction of the participant with a counselor, and a similar percentage (73%) included some form of interaction with the system. A little over half of the interventions (53%) included interaction with peers, with and without counselor interaction. The average percentage of participants who adhered to an intervention is 50.3% (min 1%; max 93%). The values of each of the variables for each included intervention can be found in Multimedia Appendix 3.

### Differences in Intervention Characteristics between Health Care Areas

When comparing the interventions of the different health care areas using Fisher’s exact tests, we find significant differences on intended usage (*P* = .004), setup (*P*< .001), updates (*P* < .001), frequency of interaction with a counselor (*P* < .001), the system (*P* = .003), and peers (*P* = .017). When looking at the standardized residuals (data not shown), we can see where these differences are manifested. We see that lifestyle interventions are more often intended to be used less than once a month than interventions in the other areas. We see that mental health interventions are less often free in terms of their setup than the other two areas. Lifestyle interventions are more often not updated or updated without a known frequency. Regarding interaction with a counselor, we see that lifestyle interventions more often do not employ this feature. Furthermore, we see that lifestyle interventions more frequently include interaction with the system less than once a week. Finally, on interaction with peers, chronic interventions more often have interaction for which the frequency is not specified. One-way analyses of variance show that there are differences in duration (F = 6.068, *P* = .004) and adherence (F = 4.833, *P* = .010). Bonferroni post hoc analyses show that the difference in duration is between lifestyle and mental health interventions (lifestyle interventions are longer), whereas the difference in adherence is between lifestyle and chronic condition interventions and between lifestyle and mental health interventions (lifestyle interventions have a lower adherence rate). In sum, lifestyle interventions are longer, the intended usage is less frequent, they have fewer updates, there is less interaction with the system and a counselor, and there is lower adherence than interventions aimed at chronic conditions and mental health. Mental health interventions are less often free in their setup and interventions aimed at a chronic condition include interaction with peers more often, for which the frequency is not specified.

### Persuasive Technology

When examining the persuasive technology elements that are presented in Table 3, we see that a mean of 5.6 (median 5) out of a possible 21 elements were used within a web-based intervention. Primary task support shows the highest mean (2.9 out of a possible 7; median 3), while social support shows the lowest mean (1.2 out of a possible 7; median 1). One-way analyses of variance show that there is a significant difference between the use of persuasive technology elements for primary task support (F = 5.631, *P* = .005). A Bonferroni post hoc analysis shows that this difference is between lifestyle and mental health interventions, where lifestyle interventions employ a higher mean of elements than mental health interventions. Furthermore, we can see that in primary task support, tunneling is used most often (n = 75; 90%), closely followed by tailoring (n = 73; 88%). Tunneling is used in all included mental health interventions, but only in 10 (63%) of lifestyle interventions (significant difference; *P* < .001). Reduction and self-monitoring are less often used in mental health interventions than in the other areas (significant difference reduction, *P* = .033; and self-monitoring, *P* < .001). This is most strikingly seen in self-monitoring, which is used in 94% of lifestyle interventions, as opposed to 12% in the mental health interventions. Overall, rehearsal and simulation are used least of all out of the primary task support elements. From the dialogue support elements, reminders are most often used (n = 61; 74%) across all areas. Suggestion is the second most frequently used element (n = 24; 29%), although this is used more often in web-based interventions targeted at chronic conditions than in mental health (*P* = .008). Praise was not used in any of the interventions and rewards were used only in 3 interventions. In social support, we see that social facilitation is most often used (n = 43; 52%), with a significant difference between interventions aimed at a chronic condition (n = 14; 74% including social facilitation) and at lifestyle (n = 5; 31%; *P* = .046). Furthermore, social learning and social comparison are used reasonably frequently (respectively n = 31; 39% and n = 14; 17%), with mental health interventions predominantly contributing to these numbers (with a significant difference only for social learning: *P* = .044). Cooperation, on the other hand, is used in 2 lifestyle interventions and 1 chronic intervention, but in none of the mental health interventions (significant difference; *P* = .041). The other elements (normative influence, competition, and recognition) are hardly used. In sum, primary task support is most extensively employed while dialogue support and social support are sparsely employed. Tunneling, tailoring (primary task support), reminders (dialogue support), and social facilitation (social support support) are the most frequently used elements. On average, lifestyle interventions employ more primary task support elements than mental health interventions.

### Predictors of Adherence

We performed a hierarchical multiple linear regression, using a block-wise “enter” method, to explore the predictors of adherence. Variables expected to predict adherence were entered in the analysis in blocks of related constructs, as specified in the methods section. The final model explained 55% of the variance in adherence. In this model, interventions studied with a RCT design (instead of an observational study), increased interaction with a counselor, more frequent intended usage, more frequent updates and more extensive employment of dialogue support significantly predicted better adherence.

**Table 2:** Descriptive variables of the included interventions per health care area

Variable		Chronic (N = 19), n (%)	Lifestyle (N = 16), n (%)	Mental (N = 48), n (%)	Total (N = 83), n (%)
Intended usage	<= 1/month	1 (5)	3 (19)	1 (2)	5 (6)
	1/month – 1/week	4 (21)	4 (25)	2 (4)	10 (12)
	1/week	13 (68)	6 (38)	40 (83)	59 (71)
	>1/week	1 (5)	3 (3)	5 (10)	9 (11)
Setup	Free	5 (26)	10 (63)	1 (2)	16 (19)
	Modular	14 (74)	6 (38)	47 (93)	67 (81)
Updates	None	1 (5)	5 (31)	1 (1)	7 (8)
	yes, FNS^a^	0 (0)	2 (13)	0 (0)	2 (2)
	<= 1/month	2 (11)	1 (6)	1 (2)	4 (5)
	1/month – 1/week	3 (16)	1 (6)	3 (6)	7 (8)
	1/week	12 (63)	6 (38)	42 (88)	60 (72)
	>1/week	1 (5)	1 (6)	1 (2)	3 (4)
Duration (weeks)	mean (sd)	18.2 (15.8)	29.8 (33.9)^b^	11.1 (18.5)	15.8 (18.5)
	Median	11	17	9	10
Interaction with counselor	None	2 (11)	8 (50)	10 (21)	20 (24)
	yes, FNS	3 (16)	3 (19)	2 (4)	8 (10)
	<1/week	5 (26)	3 (19)	2 (4)	10 (12)
	1/week	7 (37)	2 (13)	23 (48)	32 (39)
	>1/week	2 (011	0 (0)	11 (23)	13 (16)
Interaction with system	None	7 (37)	1 (6)	14 (29)	22 (27)
	yes, FNS	6 (32)	1 (6)	3 (6)	10 (12)
	<1/week	1 (5)	5 (31)	2 (4)	8 (10)
	1/week	2 (11)	6 (38)	14 (29)	22 (27)
	>1/week	3 (16)	3 (19)	15 (31)	21 (25)
Interaction with peers	none	5 (26)	10 (63)	24 (50)	39 (47)
	yes, FNS	10 (53)	4 (25)	10 (21)	24 (29)
	<1/week	2 (11)	0 (0)	1 (2)	3 (4)
	1/week	1 (5)	2 (13)	13 (27)	16 (19)
	>1/week	1 (5)	0 (0)	0 (0)	1 (1)
Face-to-face	included	3 (16)	1 (6)	1 (2)	5(6)
Phone	included	7 (37)	5 (31)	17 (35)	29 (35)
SMS	included	0 (0)	2 (13)	5 (10)	7 (8)
Adherence	mean (sd)	55.3 (19.8)	32.8 (23.0)	54.2 (27.4)	50.3 (26.2)

^a^ FNS = Frequency not specified; ^b^ Based on 13 interventions. Three interventions (23, 26, and 27) did not specify duration.

**Table 3:** Persuasive technology in web-based interventions included in this study per health care area.

Variable			Chronic (N = 19), n (%)	Lifestyle(N = 16), n (%)	Mental(N = 48), n (%)	Total(N = 83), n (%)	*P* ^a^
**Primary Task Support**		*mean (sd)*	*3.3 (1.0)*	*3.4 (1.3)*	*2.6 (1.0)*	*2.9 (1.1)*	
		*median*	*4*	*3.5*	*2*	*3*	
	Reduction		10 (53)	10 (63)	14 (29)	34 (41)	.033
	Tunneling		17 (90)	10 (63)	48 (100)	75 (90)	<.001
	Tailoring		16 (84)	14 (88)	43 (90)	73 (88)	.814
	Personalization		4 (21)	2 (13)	3 (6)	9 (11)	.209
	Self-monitoring		12 (63)	15 (94)	12 (12)	39 (47)	<.001
	Simulation		2 (11)	3 (19)	2 (4)	7 (8)	.118
	Rehearsal		1 (5)	1 (6)	0 (0)	2 (2)	.175
**Dialogue Support**		*mean (sd)*	*1.6 (1.0)*	*1.4 (1.3)*	*1.6 (0.9)*	*1.5 (1.0)*	
		*median*	*2*	*1*	*1*	*1*	
	Praise		0 (0)	0 (0)	0 (0)	0 (0)	
	Rewards		0 (0)	2 (13)	1 (2)	3 (4)	.134
	Reminders		13 (68)	11 (69)	37 (77)	61 (74)	.656
	Suggestion		11 (58)	4 (25)	9 (19)	24 (29)	.008
	Similarity		4 (21)	1 (6)	16 (33)	21 (25)	.088
	Liking		2 (11)	4 (25)	8 (17)	14 (17)	.561
	Social role		1 (5)	0 (0)	4 (8)	5 (6)	.819
**Social Support**		*mean (sd)*	*1.1 (0.7)*	*0.8 (0.9)*	*1.3 (1.2)*	*1.2 (1.0)*	
		*median*	*1*	*0.5*	*1*	*1*	
	Social learning		5 (26)	3 (19)	24 (50)	31 (39)	.044
	Social comparison		1 (5)	1 (6)	12 (25)	14 (17)	.088
	Normative influence		0 (0)	0 (0)	1 (2)	1 (1)	1.000
	Social facilitation		14 (74)	5 (31)	24 (50)	43 (52)	.046
	Cooperation		1 (5)	2 (13)	0 (0)	3 (4)	.041
	Competition		0 (0)	1 (6)	0 (0)	1 (1)	.193
	Recognition		0 (0)	1 (6)	2 (4)	3 (4)	.767
**Total**		*mean (sd)*	*6.0 (2.2)*	*5.6 (2.5)*	*5.4 (2.0)*	*5.6 (2.1)*	

^a^ Based on Fisher’s exact test. Note: results in *italics* are the mean (sd) and median number of elements used per intervention. Other results are presented as the number (%) of interventions that include a certain element.

**Table 4:** Predictors of adherence in a hierarchical multiple linear regression.

Step	Variable	*B*	*SE B*	*Beta*	*P*
1	Constant	0.40	.06		<.001
	Chronic	0.04	.07	.07	.55
	Lifestyle	-0.17	.08	-.25	.025
	Study design	0.18	.06	.30	.007
2	Constant	0.25	.09		.006
	Chronic	0.07	.07	-.11	.34
	Lifestyle	-0.11	.08	-.16	.17
	Study design	0.16	.07	.28	.014
	Freq. interaction with counselor	0.04	.02	.28	.055
	Freq. interaction with system	0.01	.02	.03	.79
	Freq. interaction with peers	0.01	.02	.05	.63
	Phone	0.09	.06	.16	.17
	Face-to-face	-0.08	.12	-.08	.48
	SMS	0.04	.10	.04	.69
3	Constant	-0.04	.21		.85
	Chronic	0.08	.07	.13	.26
	Lifestyle	-0.07	.09	-.09	.47
	Study design	0.18	.06	.30	.005
	Freq. interaction with counselor	0.02	.02	.12	.31
	Freq. interaction with system	-0.02	.02	-.09	.42
	Freq. interaction with peers	0.01	.02	.05	.60
	Phone	0.13	.06	.26	.027
	Face-to-face	-0.08	.11	-.08	.47
	SMS	0.02	.09	.03	.81
	Intended usage	0.09	.05	.23	.057
	Setup	-0.15	.11	-.22	.18
	Updates	0.10	.03	.43	.004
	Duration	-0.00	.00	-.06	.63
4	Constant	-0.12	.19		.51
	Chronic	0.08	.06	.14	.20
	Lifestyle	-0.04	.08	-.01	.96
	Study design	0.15	.06	.26	.008
	Freq. interaction with counselor	0.04	.02	.22	.039
	Freq. interaction with system	-0.04	.02	-.22	.058
	Freq. interaction with peers	-0.03	.03	-.15	.34
	Phone	0.05	.06	.10	.37
	Face-to-face	-0.10	.10	-.10	.31
	SMS	0.02	.08	.02	.85
	Intended usage	0.11	.04	.27	.014
	Setup	-0.16	.10	-.23	.11
	Updates	0.09	.03	.40	.002
	Duration	-0.00	.00	-.02	.88
	Primary task support	-0.02	.03	-.11	.41
	Dialogue support	0.09	.03	.36	.006
	Social support	0.07	.04	.27	.095

Note *R* ^2^=.14 for step 1 (*P* = .08); ∆*R* ^2^ = .10 for step 2 (*P* = .16); ∆*R* ^2^ = .15 for step 3 (*P* = .006); ∆*R* ^2^ = .15 for step 4 (*P* < .001); cumulative variance explained in the final (step 4) model: *R* ^*2*^ = .55 (*P* < .001)

## Discussion

In this systematic review, we have attempted to synthesize the combined knowledge of eHealth researchers to gain insights into the factors that affect adherence to web-based interventions in the areas of chronic conditions, lifestyle, and mental health. In this study, we viewed technology from a theoretical perspective and conceived adherence as an objective measurement that allows for comparison between different interventions.

### Principal Results

We included 101 publications describing research into 83 interventions. Mental health interventions (n = 48) constituted the largest part of these interventions. Looking at the key characteristics of web-based interventions in terms of technology and interaction, it appears that the typical web-based intervention is meant to be used once a week, is modular in setup, is updated once a week, lasts for 10 weeks, includes interaction with the system, a counselor, and peers on the web, includes some persuasive technology elements, and results in about 50% of the participants adhering to the intervention.

However, to answer our second research question, there do appear to be differences between health care areas. Overall, lifestyle interventions are longer and less strict (more employ a free setup, less frequent intended usage, fewer updates, and less interaction) than interventions aimed at chronic conditions and mental health, which seems to result in lower adherence with lifestyle interventions. Mental health interventions follow the weekly, modular format the most, with only one intervention using a free setup. This may be explained by the difference in scope of lifestyle and mental health interventions: lifestyle interventions may be more oriented towards long-term changes, while mental health interventions are often aimed at treatment that is delivered in a short, strict format. However, interventions for a chronic condition are also aimed at a long-term change or goal, but these interventions are on average more strict than lifestyle interventions. More counselor involvement is likely to be an explanation because these interventions are often offered in a health care setting and we saw a significant difference between these areas.

Regarding persuasive technology, we see that primary task support elements are most commonly employed, especially in interventions aimed at chronic conditions and lifestyle. Tunneling, which is a technological result of a modular setup, is employed most often in mental health interventions and less frequently in lifestyle interventions. This difference is a logical result of the differences in setup between interventions in these areas. This finding is not surprising, taking into account that most mental health interventions are based on regular face-to-face therapy where psycho-education and behavior modification is usually delivered step-wise (see [3]). Tailoring, which is widely recognized as an important feature of effective health communication [35, 36], is used in one form or another in 88% of the interventions. Strikingly, rehearsal, which is also seen as very important in learning and behavior change [37, 38], is seldom employed. It may be that rehearsal is seen by the authors of the articles reviewed as such an obvious part of an intervention that a description of this process is omitted from the description of the interventions. If not, this should be a point of particular interest when (re)designing web-based interventions.

Only a mean of 1.5 out of a possible 7 dialogue support elements are employed per web-based intervention. It should be noted that we have not coded the elements that may be present in email-like messages sent by a counselor because we feel that this is part of the counselor interaction and not so much a part of the dialogue support that Oinas-Kukkonen [31] and Fogg [24] describe. Reminders are the most frequently employed element. Studies have shown the importance of reminders in increasing adherence and in increasing the effectiveness of web-based interventions [7, 39]. Therefore, we found it striking that 26% of the interventions did not include reminders in some way. Suggestion was the second most frequently used element and was employed more in interventions aimed at chronic conditions than mental health. This seems likely to be due to the focus of the interventions for chronic conditions being on coping with a condition and giving suggestions or strategies to achieve this, whereas in mental health interventions the focus is often more curative to “solve” a certain problem. Praise and rewards are seldom used, which may be a shortcoming when looking at the recent literature into serious gaming and gamification, where employing game-like strategies, such as praise and rewards, are expected to have positive effects on the outcomes of health interventions [40, 41].

Social support is widely recognized as an important strategy in behavior change [42, 43] and it might be disappointing to see that, on average, only 1.2 out of a possible 7 elements are used per web-based intervention. Social facilitation was used in more than half of the interventions. It must be noted that here social facilitation means providing the opportunity to contact others using the same intervention; it does not say anything about whether the opportunity is actually used. In practical terms, this means that when an intervention includes a discussion board, social facilitation is employed, even when there are no posts on the discussion board. Social learning and social comparison were employed through, for example, obligatory posts of exercise answers on a discussion board or by providing a story by a user (real or fictive), including how he or she dealt with the situation. Cooperation, competition, normative influence, and recognition are seldom used and therefore provide areas in which web-based interventions might be improved. However, in this study, social support did not affect adherence, so more research is needed to investigate whether or not this area provides added value.

Our third research question was about the percentage of participants that adhere to web-based interventions. We found an average adherence of 50%, which confirms that non-adherence is an issue in web-based interventions. There was a wide range in the level of adherence, with 6 interventions scoring below 10% adherence and 5 interventions scoring 90% adherence or higher. Our last research question was aimed at determining which characteristics of web-based interventions relating to technology and interaction are related to better adherence. Using a hierarchical multiple linear regression, our final model explains 55% of the variance in adherence, which, in our view, is a substantial amount that provides valuable insights into the issue of adherence.

Interestingly, the first two models (including the context of the intervention and the interaction within the intervention) were not significant. It was only when aspects relating to the format of the intervention and the technology employed were entered that the model reached significance. In the final model, an RCT, as opposed to an observational study, significantly predicted better adherence. A likely explanation is that the observational studies in our review were mainly small pilot studies and large real-life studies. Pilot studies are likely to show lower adherence rates because the interventions are not fully tested and are improved after the outcomes of the pilot are known. Real-life observational studies have been shown to have lower adherence rates, which suggests that the formal structure of a trial is important for participants to adhere to an intervention [34]. Furthermore, the selection processes of many RCTs make it likely that there is a difference in the participants in both settings, which contributes to the difference in adherence.

The frequency of interaction with a counselor was a significant predictor of adherence. This finding concurs with reviews of Brouwer [29], Schubart [30], and other studies (for an overview see [44]) that conclude that counselor or clinician support is related to greater exposure and engagement. Of the significant predictors in our study, this variable contributes the least. In our review, we have found no evidence that the frequency of interaction with peers is related to adherence. This is somewhat contrary to the results of Brouwer [29], who concluded that peer support was related to greater exposure. In that study, exposure was seen as the time visitors spend on the website, which is very different from our definition of adherence. Furthermore, in this study, we coded the frequency of interaction, not merely whether there was any interaction or not. This resulted in 29% of interventions being coded as, “Yes, there is interaction with peers, but the frequency is unknown.” This frequency may vary to a large degree between these interventions, but without clear information we cannot make a distinction, which may have influenced our results.

In the final model, the frequency of interaction with the system seems to negatively influence adherence, although not significantly. This surprising finding may be explained by the fact that more interaction with the system meant, in many cases, that there was no interaction with a counselor. More frequent intended usage also predicts better adherence. This might seem counterintuitive, but might also mean that when people are expected to be more active they become more engaged with the system. Moreover, more frequent intended usage will, in many cases, lead to more frequent reminders and we know that reminders can positively influence adherence [39]. That the provision of frequent updates is important was also seen in the review of Brouwer [29] and is confirmed in this study.

Finally, more extensive employment of dialogue support is related to better adherence. This outcome was predicted by the persuasive system design model [31], but this study is, to our knowledge, the first to confirm this outcome related to adherence in a health setting. When looking at the other persuasive technology categories, we see that social support shows a trend towards a significant contribution to better adherence. We feel that this trend warrants further investigation. It might be that it has no significant predictive value in this study because of the limited use of social support elements in the included interventions. Interestingly, primary task support does not show any predictive value for adherence. This may well be explained by the purpose of the employment of primary task support. As indicated in the name, these elements make the primary task (ie, the goal of the intervention) easier, and are not so much focused on the process (ie, using the intervention or adhering to the intervention). It seems likely that these elements play a more important role in the effect of the intervention than in the adherence.

A final comment on the model for the prediction of adherence is on the different health care areas. We see that in the first model, lifestyle interventions, as opposed to mental health interventions, predict a lower adherence, but when adding the characteristics of the interventions in the model, this predictive value is negated. It seems that the health care area *per se* does not predict adherence, but the differences in the characteristics of the interventions in these areas do predict adherence.

### Implications and Recommendations

Taking into account the results of this study, it seems reasonable to not only hope for adherence, but to plan for adherence when designing web-based interventions. Although 33 studies that are included in this review state that they have planned for adherence, it is remarkable that 18 state that encouraging adherence is a task for the counselor [45-62] and one study included monetary incentives to promote adherence [63]. Of the 15 studies that mention adapting the design of the intervention to increase adherence, 8 studies do so without any theoretical basis or reference [64-71], 4 studies make the adaptation the focus of their study [72-75], and 2 studies have adapted the design based on a prior study on the same intervention [76, 77]. Overall, it seems that adapting web-based interventions to promote adherence is done in an ad-hoc manner and that a framework to guide researchers and developers in this area is needed. The PSD model [31] may provide such a framework for the design of web-based interventions.

Moreover, it seems valuable to look much further than the health care area for which the intervention is being designed. Although each health care area has its own demands and limitations, the different areas might learn from each other’s strong points. Lifestyle interventions, although aimed at long-term goals, might benefit from incorporating segments with a more strict format and shorter duration. Mental health interventions might be extended to aim at more long-term goals like relapse prevention. They may therefore employ a less strict format, while being aware that adherence might become a larger problem. Moreover, mental health interventions might include the primary task support elements used in chronic condition and lifestyle interventions.

Furthermore, we now have evidence that certain intervention characteristics and persuasive technology can improve adherence. It seems that expecting a certain amount of engagement from the target group can actually be helpful in promoting adherence and is something that seems to be easy to implement in new and existing web-based interventions. We must keep in mind that the effect of intended usage might also be due to a bias among the participants when only those participants who agree in advance with a high level of engagement participate in such interventions. Duration seems harder to change. Cutting an intervention into shorter segments may be enough to improve adherence, but this should be investigated further. Including and possibly increasing the frequency of interaction with a counselor seems a more costly way to improve adherence and might, therefore, be a less than optimal starting point when specifically used as a strategy to increase adherence. Increasing dialogue support using persuasive technology seems to be a more cost-effective vantage point in this respect and may even be enhanced by the increasing use of mobile technology, which seems likely to, in turn, offer a valuable platform for introducing on-the-spot reminders and feedback.

Additionally, our results can be of value for blended care (ie, a combination of online and face-to-face care) by clarifying the crucial aspects for promoting adherence in web-based interventions. When it is not possible to adapt a web-based intervention to promote adherence, it may be feasible to include a face-to-face segment in the overall intervention at a crucial stage to make up for the predicted loss of adherence.

The results of this study can be used to make an informed decision about how to design a web-based intervention that has a greater likelihood of patient adherence. It must be noted, however, that we do not advocate a so-called “technology push” where technology is introduced only for the sake of the technology and the ability to create the technology. It should always be created in close collaboration with the target audience and with a clear goal to create a viable eHealth technology [12]. This study provides insights into the choices one can make with the target audience.

In this study, we defined adherence as being the proportion of participants who use the intervention as it is intended to be used. By doing this, we have created an adherence measurement from objective data that is comparable between interventions. We feel that the study shows that this is a promising approach and this adherence measurement can be used for a wide variety of studies. However, to date, few studies report adherence as the measurement we have chosen to use. For review studies, this means that researchers have to define the intended use, search for the usage data that corresponded to this intended use, and then calculate the adherence. This might lead to a different interpretation of the usage data than the original authors intended. However, from our experience, we can say that as long as there is enough information on the intervention and the usage, it is feasible to calculate an objective and comparable adherence measurement. For intervention studies, we would advise researchers to at least provide the information needed (ie, intended usage and usage data related to this intended usage) to calculate this adherence measurement and, preferably, to state the calculated adherence percentage for easy comparison between interventions.

### Limitations

In this study, we have excluded many interventions because data about usage was absent or the usage data that was presented had no direct relationship to the intended use. For example, we excluded studies that only presented mean login data per week for all respondents and had an intended usage of once a week because these data do not show us which percentage of respondents logged in each week. This strict selection based on usage data might have introduced a bias in our included studies.

We have coded the web-based interventions included in this study based on the descriptions in the published literature. Although we have made an effort to find all the information in the published literature about each intervention, our coding was limited by the description of the interventions on paper. As is noted by other authors, the description of these interventions is varied [12, 29, 30], which makes it difficult to capture all the characteristics of each intervention, and this might have influenced our results. Initiatives to standardize and improve the description of web-based interventions like the consort statement for eHealth [12], a protocol for systematic reviews in eHealth [33], and guidelines for executing and reporting internet intervention research [78] are therefore very necessary and will hopefully improve the possibility to compare eHealth technologies and learn from each other.

Lastly, a limitation of this review might be that we have only focused on the published literature. We have not included grey literature and have therefore included little real-life adherence data. As noted by Christensen [34], there is a difference between the usage of web-based interventions in a research setting and in a more real-life setting. We have tried to cope with this by using a strict definition of adherence, separating it from following the research protocol and filling out questionnaires, and by coding all interaction that might be the result of being part of a study as part of the intervention. Nonetheless, the limited amount of real-life data in our review might have influenced the results.

Overall, our results confirm the conclusions of prior studies [29, 30] that interaction with a counselor and regular updates promote adherence. Furthermore, the results of this review elaborate on the role of intervention characteristics (duration, setup, and intended usage) and persuasive technology, especially elements to support the dialogue. Finally, this study has provided practical recommendations to increase adherence when (re)designing a web-based intervention.

### Future Research

The data and results from this study provide numerous points of departure for future research. To increase our understanding of the characteristics of web-based interventions and their effect on adherence, it would be interesting to compare interventions that show high adherence with interventions that show low adherence using in-depth, qualitative analyses. The positive deviance approach used by Schubart [30] seems appropriate for this goal. Furthermore, it is interesting to test our statistical adherence model in experimental studies. Additionally, expanding the model by including the characteristics of participants seems to be relevant. Finally, exploring the relationship between persuasive technology, especially primary task support, and (clinical) outcomes of an intervention is likely to be a worthwhile line of research.

## Multimedia Appendix 1

Keywords literature search.

## Multimedia Appendix 2

Included interventions, targeted behavior or conditions, and studies.

## Multimedia Appendix 3

Characteristics of, and adherence to, web-based interventions included in this study.
