# Supplementary material for: Persuasive System Design Does Matter: A Systematic Review of Adherence to Web-Based Interventions
Source: J Med Internet Res. 2012 Nov 14;14(6):e152. doi: 10.2196/jmir.2104 (PMC3510730; doi:10.2196/jmir.2104)
Supplement: Supplementary file 1 [file jmir_v14i6e152_app1.pdf]

Multimedia Appendix 1. Keywords literature search

**Web-based**	**Intervention**	**Adherence**	**Health**
web page	treatment	Motiv*	health*
web application	intervention	attrition	behavio*
website	program	dropout	manage*
internet delivered	programme	drop out	self help
web based	therapy	adherence	self control
internet based	coach	nonadherence	selfmanagement
internet mediated		compliance	self care
internet supported		noncompliance	
Online*		Persist*	
Medical informatics		Response*	
Information technology		nonresponse	
e health		loyalty	
ehealth		engagement	
e therap*		disengagement	
Telemedic*		involvement	
telecare		noninvolvement	
telehealth		reach	
e mental health		Intention*	
Emental health		Satisfy*	
