# Supplementary material for: Persuasive System Design Does Matter: A Systematic Review of Adherence to Web-Based Interventions
Source: J Med Internet Res. 2012 Nov 14;14(6):e152. doi: 10.2196/jmir.2104 (PMC3510730; doi:10.2196/jmir.2104)
Supplement: Supplementary file 2 [file jmir_v14i6e152_app2.pdf]

Multimedia appendix 2. Included interventions, targeted behavior or conditions, and studies

**Intervention name**	**Behavior/Condition**	**Studies**
Chronic Condition		
1. Van den Berg	arthritis	Van den Berg 2007 [45]; Van den Berg 2006 [79]
2. Teens Taking Charge	arthritis	Stinson 2010 [80]
3. Rheumates@work	arthritis	Lelieveld 2010 [81]
4. Oneself	chronic pain	Schulz 2010 [82]
5. WebMAP	chronic pain	Long 2009 [83]; Palermo 2009 [84]
6. SPAIN	CVD	Goessens 2008 [59]; Goessens 2006 [85]
7. DPP	diabetes	McTigue 2009 [60]
8. NetPLAY	diabetes	Liebreich 2009 [71]
9. My Path1	diabetes	Glasgow 2011 [70]
10. My Path2	diabetes	Glasgow 2011 [70]
11. YourWay	diabetes	Mulvaney 2010 [86]
12. 'Diabetergestemd'	diabetes	Van Bastelaar 2011a [77]; Van Bastelaar 2011b [87]
13. WebEase	epilepsy	DiIorio 2009 [88]
14. Rekindle	erectile dysfunction	McCabe 2008 [61]; McCabe 2009 [89]
15. Heartnet	heart transplant	Dew 2004 [90]
16. Ljottson	IBS	Ljottson 2010 [58]
17. MyMigraine	migraine	Sorbi 2010 [91]
18. EPP online	self-management	Lorig 2008 [92]; Lorig 2006 [62]
19. Andersson-T	tinnitus	Andersson 2002 [93]
Lifestyle		
20. 5 a Day, the Rio Grande Way	nutrition	Woodall 2007 [74]; Buller 2008 [68]
21. Active U	PA	Buis 2009 [94]
22. Fun, Food and Fitness Club	PA, nutrition	Thompson 2008 [75]; Thompson 2007 [95]; Baranowski 2003 [96]
23. Healthy Life Check	PA, nutrition, smoking cessation	Brouwer 2010 [97]
24. Happy Ending	smoking cessation	Brendryen 2008a [98]; Brendryen 2008b [99]
25. Lenert	smoking cessation	Lenert 2003 [100]
26. QuitCoach	smoking cessation	Balmford 2008 [101]
27. QuitNet	smoking cessation	Graham 2007 [102]; Saul 2007 [103]; Cobb 2005 [104]
28. Real U	smoking cessation	An 2008 [63]; An 2006 [105]
29. Health Partners	weight management	Van Wier 2009 [57]
30. Healthy Weight for Life	weight management	Cussler 2008 [69]
31. LEARN	weight management	Hunter 2008 [106]
32. Weight Loss Management	weight management	Meenan 2009 [107]; Svetkey 2009 [108]; Stevens 2008 [76]; Funk 2010 [109]
33. Step up, Trim down	weight management	Bennet 2010 [67]
34. Healthy Weight Assistant	weight management	Kelders 2011 [11]
35. SHED-IT	weight management	Morgan 2011 [110]
Mental Health		
36. BRAVE1	anxiety	March 2009 [66]
37. BRAVE2	anxiety	Spence 2011a [64]
38. Worry Program	anxiety	Titov 2009a [111]
39. Anxiety program	anxiety	Titov 2010a [112]
40. Andersson-A	anxiety	Andersson 2011 [113]
41. Hedman	severe health anxiety	Hedman 2011a [50]
42. Down Your Drink	alcohol	Linke 2007 [114]; Linke 2004 [115]
43. 'Alcohol de Baas'1	alcohol	Postel 2010a [116]
44. 'Alcohol de Baas'2	alcohol	Postel 2010b [117]
45. M-PASS	alcohol	Bingham 2010 [118]

46. MORE	alcohol and drugs	Klein 2012 [119]
47. RealTeen	drug abuse prevention	Schwinn 2010 [120]
48. BEP1	bipolar disorder	Nicholas 2010 [121]
49. BEP2	bipolar disorder	Nicholas 2010 [121]
50. Everything under Control	depression	Van Straten 2008 [122]; Warmerdam 2008 [123]
51. Colour your Life1	depression	De Graaf 2009a [124]; De Graaf 2009b [125]
52. Colour your Life2	depression	Warmerdam 2008 [123]
53. Deprexis	depression	Meyer 2009 [126]
54. Master your Mood	depression	Gerrits 2007 [51]
55. MoodGym	depression	Christensen 2006 [127]
56. Sadness	depression	Perini 2009 [52]
57. MoodManager	depression	Mohr 2010 [46]
58. Vernmark	depression	Vernmark 2010 [47]
59. Wellbeing program	depression and anxiety	Titov 2011 [128]
60. Carrard1	eating disorder	Carrard 2011a [129]
61. Carrard2	eating disorder	Carrard 2011b [48]
62. Carlbring1	panic disorder	Carlbring 2005 [130]
63. Carlbring2	panic disorder	Carlbring 2006 [131]
64. Panic Center	panic disorder	Farvolden 2005 [132]
65. Panic Online1	panic disorder	Klein 2006 [133]; Richards 2006 [134]
66. Panic Online2	panic disorder	Richards 2006 [134]; Kiropoulos 2008 [135]; Klein 2009 [136]
67. Panic Online3	panic disorder	Klein 2009 [136]
68. Interapy	panic disorder	Ruwaard 2010 [137]
69. Panic program	panic disorder	Wims 2010 [138]
70. PTSD program	PTSD	Spence 2011b [49]
71. PTSD online	PTSD	Klein 2010 [139]
72. Andersson-S1	social phobia	Andersson 2006 [140]; Tilfors 2008 [141]
73. Andersson-S2	social phobia	Tilfors 2008 [141]; Furmark 2009 [142]; Tilfors 2011 [143]
74. Andersson-S3	social phobia	Carlbring 2007 [144]
75. Andersson-S4	social phobia	Hedman 2011b [145]
76. IAR	social phobia	Furmark 2009 [142]
77. Shyness1	social phobia	Titov 2008a [55]; Titov 2008b [56]; Titov 2008c [54]
78. Shyness2	social phobia	Aydos 2009 [53]
79. Shyness3	social phobia	Titov 2008c [54]
80. Shyness4	social phobia	Titov 2009b [72]
81. Shyness5	social phobia	Titov 2009b [72]; Titov 2009c [73]
82. Shyness6	social phobia	Titov 2009c [73]
83. Shyness 7	social phobia	Titov 2010b [65]
