# Supplementary material for: Persuasive System Design Does Matter: A Systematic Review of Adherence to Web-Based Interventions
Source: J Med Internet Res. 2012 Nov 14;14(6):e152. doi: 10.2196/jmir.2104 (PMC3510730; doi:10.2196/jmir.2104)
Supplement: Supplementary file 3 [file jmir_v14i6e152_app3.pdf]

Multimedia appendix 3. Characteristics of, and adherence to, web-based interventions included in this study

**Intervention name** ***study level***	**Intended usage**	**Actual usage**	**Setup**	**Updates**	**Duration (weeks)**	**Interaction**[a]			**Moda-lity**[b]			**Persuasive technology**[c]			**Adherence**
						System	Care provider	Peers	1	2	3	PTS	DS	SS	(in %)
Chronic Condition															
1. Van den Berg *RCT*	return PA schedule at least 26/52 weeks	n = 44 high or sufficient PA schedule return rate	modules	weekly	52	none	1/week	FNS	+	-	-	b,c	k	r	52
2. Teens Taking Charge *RCT*	complete 12 modules and weekly phone calls with a coach within a maximum of 21 weeks	91% completed 12 modules	modules	weekly	12-21	none	more	FNS	-	-	+	b,c, e	j	r	91
3. Rheumates @work *RCT*	complete 17 lessons in 17 weeks	n = 14 completed the internet program	modules	weekly	17	FNS	1/week	less	+	-	+	b,c, d,f	j,k, m,n	r	82
4. Oneself *Observational*	login 11 times in 12 months	n = 516 used 0-10 times; n = 169 used 11-100 times; n = 63 used 101+ times	free	weekly	52	none	less	FNS	-	-	-	a,c	-	r	31
5. WebMAP *RCT*	complete 8 modules	20/26 completed all 8 modules	modules	weekly	8	more	1/week	none	-	-	-	b,c, d,e, g	j,l, m	p	77
6. SPAIN *Observational*	login frequently	n = 5 used <6 times; n = 13 used >=6 - <26 times; n=18 used >=26 -	free	weekly	26	FNS	less	none	-	-	-	a,c, e	j	-	64

		<41 times; n=14 used >=41 times													
7. DPP *Observational*	complete 24 lessons	n = 8 completed 21-24 lessons	modules	biweekly	52	less	less	1/wee k	-	-	+	a,b, c,e	j,k	r	16
8. NetPLAY *RCT*	once a week for at least 8 weeks	n = 15 used at least once per week for a minimum of 8 weeks	modules	weekly	12	FNS	1/week	FNS	-	-	-	a,b, c,e	j,k	r	60
9. My Path1 *RCT*	login weekly	66% logged in weekly from 0 to 6 weeks; 44% logged in weekly from 6 weeks to 4 months	free	once in 4 months	16	more	FNS	FNS	-	-	+	a,b, c,e	j,k	r	44
10. My Path2 *RCT*	login weekly	74% logged in weekly from 0 to 6 weeks; 41% logged in weekly from 6 weeks to 4 months	free	once in 4 months	16	more	less	less	+	-	+	a,b, c,e	j,k	o,r	51
11. YourWay *RCT*	view 6 stories in 11 weeks	63% viewed all 6 stories	modules	biweekly	11	1/week	FNS	FNS	-	-	-	b,c, d,f	j,k,l	p,r	63
12. Diabetergestem d *RCT*	complete 8 lessons	n = 53 completed all 8 lessons	modules	weekly	8	1/week	1/week	FNS	-	-	-	a,b, c,e	j,l	o,r	42
13. WebEase *Observational*	complete 3 biweekly modules	n = 15 completed all 3 modules	modules	daily	6	FNS	none	FNS	-	-	-	a,b, c,e	-	r	43
14. Rekindle *RCT*	complete 5 modules in 10 weeks	n = 12 completed all 5 modules	modules	biweekly	10	none	less	none	-	-	-	b,e	j	-	30
15. Heartnet	at least weekly	n = 10 used	free	none	17	none	FNS	FNS	-	-	+	a,b	k	r	42

Observational		weekly or more often													
16. Ljottson *RCT*	complete 5 steps in 10 weeks	n = 29 reached fifth step and engaged in exposure exercises	modules	weekly for first 5 weeks	10	none	weekly	FNS	-	-	+	b,c	j	r	74
17. MyMigraine *Observational*	complete 8 lessons in 10 weeks	n = 6 completed 8 lessons	modules	weekly	10	FNS	none	none	-	-	-	b,e	k,l	o	60
18. EPP online *Observational*	login several times a week	79% reached last session	modules	weekly	6	FNS	more	more	-	-	-	a,b, c,e	j,k	o,r,s	79
19. Andersson-T *RCT*	complete 6 modules	n = 26 finished treatment	modules	weekly	6	none	1/week	none	-	-	-	b,c, e	k	-	49
Lifestyle															
20. 5 a Day, the Rio Grande Way *RCT*	Login once a month	192 used at least once; of these mean login = 3.3; 75th percentile = 4; n = 48 used at least 4 times	free	monthly	17	less	none	none	-	-	-	a	k	-	13
21. Active U *Observational*	Login and enter monitoring data once a week	n = 2304 entered data for all 8 weeks of the program	free	weekly	8	1/week	none	FNS	-	-	-	c,e	j,k	s,t	31
22. Fun, Food and Fitness Club *RCT*	Login once a week and complete activities	Study 1 (I = 19): lowest logon % per week = 37%; Study 2 (I = 78) lowest logon % per week = 68%	modules	weekly	8	1/week	weekly	none	-	-	+	b,c, e,f	i,j,l, m	o,s	63
23. Healthy Life Check	login multiple times	n = 4857 visited more	free	none	NS	FNS	none	none	-	-	-	b,c, e	-	-	6

Observational		than once													
24. Happy Ending *RCT*	400 contact moments	Study 1: n = 45 quit the intervention; Study 2: n = 57 quit the intervention	modules	daily	54	more	none	none	-	+	+	a,b, c,e	j,k	-	70
25. Lenert *Observational*	complete 8 modules	n = 10 completed all modules	modules	weekly	8	1/week	none	none	-	-	-	a,b, c,e	j	-	20
26. QuitCoach *Observational*	Login at least 5 times	3% used at least 5 times	free	none	NS	less	none	none	-	-	-	a,b, c,e	j	-	3
27. QuitNet *Observational*	Login at least 4 times	Study 1: n = 425 used at least 4 times; Study 2: n = 115 used at least 4 times	free	none	NS	less	FNS	FNS	-	-	-	a,c, e,f	j	r	23
28. Real U *RCT*	20 weekly visits	n = 172 visited weekly	modules	weekly	30	1/week	none	1/wee k	-	-	-	b,c, e	j	o,u	67
29. Health Partners *RCT*	complete 10 biweekly modules	18% was counseled for 10 modules	modules	biweekly	20	less	less	none	-	+	-	a,b, c,e	j	-	18
30. Healthy Weight for Life *RCT*	Login and enter data once a week	39% used diet log at least weekly	free	FNS	52	none	FNS	1/wee k	-	-	-	a,e	-	o,r	39
31. LEARN *RCT*	5 logins a week	Mean login 2 per week; 4th quartile 133.6±83.7 logins; n = 57 used at least 120 times	modules	weekly	24	1/week	1/week	none	-	-	+	b,c, e	-	-	25
32. Weight Loss Management *RCT*	login once a month	n = 212 logging in and having at least one weight entry for 26/28	free	weekly	130	1/week	FNS	FNS	-	-	+	a,b, c,d, e,f,g	i,j,k ,m	r	61

		months												
33. Step up, Trim down *RCT*	using the website at least 3 times weekly	at least n = 22 met login goal each week	free	none	12	more	less	none	+	-	+	a,c, e	j,m	p,r	43
34. Healthy Weight Assistant *RCT*	biweekly visits	n = 4 logged in 6 times or more	free	FNS	12	less	none	none	-	-	-	b,c, d,e	j	-	3
35. SHED-IT *RCT*	at least 7 weeks of submission of daily eating and exercise diaries and weekly check-ins	n = 14 complied	free	none	12	more	less	FNS	-	-	-	a,c, e	m	r	41
Mental Health															
36. BRAVE1 *RCT*	complete 10 weekly sessions	n = 13 completed all sessions	modules	weekly	10	more	1/week	none	-	-	+	b,c	j,k, m	-	32
37. BRAVE2 *RCT*	finish 10 sessions	39% finished 10 modules	modules	weekly	12	more	1/week	none	-	-	+	b,c, d,e,f	j,l, m	o	39
38. Worry Program *RCT*	complete 6 lessons in 9 weeks	n = 18 finished all lessons	modules	weekly	9	more	1/week	FNS	-	-	+	b,c	j,n	o,r	72
39. Anxiety program *RCT*	complete 6 lessons in 8 weeks	n = 30 completed all 6 lessons	modules	weekly	8	1/week	1/week	FNS	-	-	-	b,c	j,l	o,r	75
40. Andersson-A *Observational*	complete 10 self-chosen modules	n = 9 completed 10 modules	modules	weekly	10	none	1/week	none	-	-	-	b,c, d	j	-	33
41. Hedman *RCT*	complete all 12 modules	n = 14 completed 12 modules	modules	weekly	12	none	1/week	FNS	-	-	-	b,c	j	r	35
42. Down Your Drink *Observational*	complete 6 lessons	Study 1: n= 79 completed all lessons; Study 2: n = 1654 completed all lessons	modules	weekly	6	more	none	FNS	-	+	-	a,b, c,e,f	j,k	r	15

43. Alcohol de Baas1 *Observational*	complete 12 week program	n = 173 completed treatment	modules	weekly	12	more	more	FNS	-	-	-	a,b, c,e	j	r	21
44. Alcohol de Baas2 *RCT*	complete 12 week program	n= 36 completed treatment	modules	weekly	12	more	more	FNS	-	-	-	a,b, c,e	j	r	46
45. M-PASS *RCT*	complete 4 online sessions	80% completed all 4 sessions	modules	weekly for 4 weeks	9	more	none	none	-	-	-	a,b, c,e	j,l, m,n	q	80
46. MORE *Observational*	finish 7 modules in 18 months	n = 58 accessed all 7 modules	modules	7 times in 18 months	78	less	less	FNS	-	-	+	a,b, c,e	j,k, m	r,u	5
47. RealTeen *RCT*	finish 12 modules in 6 weeks	n = 108 completed all 12 sessions	modules	twice weekly	6	more	none	FNS	-	-	-	a,b, c,d	i,l, m,n	o,p, r	92
48. BEP1 *RCT*	complete 8 modules	n = 80 completed 8 modules	modules	weekly	8	1/week	none	none	-	-	-	b	j,m	-	67
49. BEP2 *RCT*	complete 8 modules	n = 80 completed 8 modules	modules	weekly	8	1/week	FNS	none	-	-	-	b,c	j,m	o	81
50. All under Control *RCT*	complete 4 lessons	Study 1: n= 59 completed whole course; Study 2: n = 33 completed whole course	modules	weekly	5	1/week	1/week	none	-	-	-	a,b, c	j	o	47
51. Colour your Life1 *RCT*	complete 8 sessions and a booster sessions 12 weeks later	n = 14 completed 8 sessions	modules	weekly	9	1/week	none	none	-	-	-	b,e	-	-	14
52. Colour your Life2 *RCT*	complete 8 sessions and a booster sessions 12 weeks later	n = 34 completed whole course	modules	weekly	9	1/week	1/week	none	-	-	-	a,b, c	j	-	39
53. Deprexis	complete 9/12	n = 46	free	none	9	FNS	none	none	-	-	-	b,c	k,n	-	14

RCT	modules	completed 9 modules													
54. Master your Mood *Observational*	complete 8 lessons	n = 50 completed all sessions	modules	weekly	8	none	more	1/wee k	-	+	-	b,c	j	p,r	26
55. MoodGym *RCT*	complete 5 modules	n = 9 completed 5 modules	modules	weekly	6	none	none	none	-	-	-	b	l	-	2
56. Sadness *RCT*	complete 6 lessons in 8 weeks	n = 20 completed all lessons	modules	weekly	8	none	1/week	1/wee k	-	-	+	b,c	j,l	o,p, r	74
57. MoodManager *Observational*	complete 6 learning modules and fill out self-management tools daily	n = 19 completed 6 modules	modules	weekly	6	more	more	none	-	-	+	a,b, c,e	j,k, m	-	91
58. Vernmark *RCT*	finish all 7 modules in time	n = 17 finished in time	modules	weekly	8	1/week	1/week	none	-	-	-	b,c	j	-	59
59. Wellbeing program *RCT*	complete 8 lessons in 10 weeks	n = 30 completed all 8 lessons	modules	weekly	10	more	1/week	1/wee k	-	-	+	b,c	j,k,l	o,p, r,u	81
60. Carrard1 *Observational*	complete 7 modules	n = 40 completed all 7 modules	modules	biweekly	16	FNS	1/week	none	-	-	-	a,b, c,e	l	o	31
61. Carrard2 *RCT*	complete 11 modules in 6 months	n = 25/74 completed 11 modules	modules	biweekly	26	FNS	1/week	none	-	-	+	a,b, c,e	j	-	34
62. Carlbring1 *RCT*	complete 10 lessons and post in the bulletin board	28% finished all modules within the timeframe	modules	weekly	10	1/week	1/week	1/wee k	-	-	-	b,c	-	o,r	28
63. Carlbring2 *RCT*	complete 10 lessons and post in the bulletin board	80% finished all modules within the timeframe	modules	weekly	10	1/week	more	1/wee k	-	-	+	b,c	j	o,r	80
64. Panic Center *Observational*	complete 12 lessons	n = 12 completed the	modules	weekly	12	1/week	FNS	FNS	-	-	-	b,e	-	r	1

		12 week program													
65. Panic Online1 *RCT*	complete 6 modules	Study 1: n= 18 completed all modules; Study 2: n = 10 completed all modules	modules	weekly	8	none	more	none	-	-	-	a,b, c	-	-	90
66. Panic Online2 *RCT*	complete 8 modules	Study 1: n= 10 completed all modules; Study 2: n = 41 completed all modules; Study 3: n = 22 completed all modules	modules	weekly	12	none	more	none	-	-	-	a,b, c	j	-	86
67. Panic Online3 *RCT*	complete 8 modules	n= 21 completed all modules	modules	weekly	12	none	1/week	none	-	-	-	a,b, c	j	-	72
68. Interapy *RCT*	complete 7 modules in 11 weeks	n = 47/58 completed treatment Note: waiting list had access to intervention after intervention period	modules	at least biweekly	11	none	more	none	-	-	-	b,c, e	k	-	81
69. Panic program *RCT*	complete all 6 lessons	n = 23 completed all 6 lessons	modules	biweekly	8	none	less	less	-	-	+	b,c	j,l	o,p, r	79
70. PTSD program *RCT*	complete all 7 lessons in 8 weeks	n = 18 completed all 7 lessons	modules	weekly	8	more	1/week	FNS	-	-	+	b,c	j,k	o,r	78
71. PTSD online *Observational*	complete 10 modules in 10 weeks	n = 16 completed the program	modules	weekly	10	none	1/week	none	-	-	-	b,c	k	-	73

72. Andersson-S1<br>*RCT*	complete 9 modules + post on bulletin board once a week	Study 1: n= 8 completed all modules; Study 2: n = 18 completed all modules	modules	weekly	9	1/week	1/week	1/week	+	-	+	b,c	j	o,r	51
73. Andersson-S2<br>*RCT*	complete 9 modules + post on bulletin board once a week	Study 1: n= 10 completed all modules; Study 2: n= 37 completed all modules; Study 3: n= 0 completed all modules	modules	weekly	9	1/week	1/week	1/week	-	-	+	b,c	j	o,r	48
74. Andersson-S3<br>*RCT*	complete 9 modules + post on bulletin board once a week	n= 27 completed all modules	modules	weekly	9	1/week	more	1/week	-	-	+	b,c	j	o,r	93
75. Andersson-S4<br>*RCT*	complete 15 modules	n = 19 completed all modules	modules	weekly	15	1/week	1/week	none	-	-	-	b,c	j	-	30
76. IAR<br>*RCT*	complete 9 modules + post on bulletin board once a week	n = 10 completed all modules	modules	weekly	9	1/week	1/week	1/week	-	-	-	b,c	j	o,r	34
77. Shyness1<br>*RCT*	complete 6 lessons and post on bulletin board	Study 1: n= 39 completed all lessons; Study 2: n = 33 completed all lessons; Study 3: n = 24 completed all lessons	modules	weekly	10	none	more	1/week	-	-	+	b,c	j,l	o,p,r	79
78. Shyness2<br>*Observational*	complete 6 lessons and post on bulletin board	n = 14 completed all lessons	modules	weekly	8	none	more	1/week	-	-	+	b,c	j,l	o,p,r	74

79. Shyness3 *RCT*	complete 6 lessons and post on bulletin board	n= 10 completed all lessons	modules	weekly	10	none	none	1/wee k	-	-	-	b	l	o,p, r	33
80. Shyness4 *RCT*	complete 6 lessons	n= 56 completed all lessons	modules	weekly	8	more	none	none	-	+	-	b,c	j,l	o,p	67
81. Shyness5 *RCT*	complete 6 lessons	Study 1: n= 66 completed all modules; Study 2: n = 34 completed all modules	modules	weekly	8	more	1/week	none	-	+	+	b,c	j,l	o,p	78
82. Shyness6 *RCT*	complete 6 lessons and post on bulletin board	n= 31 completed all lessons	modules	weekly	8	more	1/week	1/wee k	-	+	-	b,c	j,l	o,p, r	76
83. Shyness 7 *RCT*	complete all 8 lessons in 11 weeks	n = 71 completed all 8 lessons	modules	weekly	11	more	none	none	-	-	-	b,c	j,l	o,p	64

[a] FNS = Frequency Not Specified; less = less than once a week; more = more than once a week; [b] 1 = face to face; 2 = SMS; 3 = phone; [c] PTS = Primary Task Support; DS = Dialogue Support; SS = Social Support; a = Reduction; b = Tunneling ; c = Tailoring; d = Personalization; e = Self-monitoring; f = Simulation; g = Rehearsal; h = Praise; i = Rewards; j = Reminders; k = Suggestion; l = Similarity; m = Liking; n = Social role; o = Social learning; p = Social comparison; q = Normative influence; r = Social facilitation; s = Cooperation; t = Competition; u = Recognition
